# The New Haplotypes of *Bartonella* spp. and *Borrelia burgdorferi* Sensu Lato Identified in *Lipoptena* spp. (Diptera: Hippoboscidae) Collected in the Areas of North-Eastern Poland

**DOI:** 10.3390/pathogens11101111

**Published:** 2022-09-28

**Authors:** Joanna Werszko, Magdalena Świsłocka, Joanna Witecka, Tomasz Szewczyk, Żaneta Steiner-Bogdaszewska, Konrad Wilamowski, Marek Asman

**Affiliations:** 1 Witold Stefański Institute of Parasitology, Polish Academy of Sciences, Twarda 51/55, 00-818 Warsaw, Poland; 2 Department of Zoology and Genetics, Faculty of Biology, University of Białystok, Ciołkowskiego 1J, 15-245 Białystok, Poland; 3 Department of Parasitology, Faculty of Pharmaceutical Sciences in Sosnowiec, Medical University of Silesia, Jedności 8, 41-218 Sosnowiec, Poland; 4 Institute of Forest Sciences, Faculty of Civil Engineering and Environmental Sciences, Białystok University of Technology, Wiejska 45e, 15-351 Białystok, Poland

**Keywords:** deer keds, *Borrelia burgdorferi* sensu lato, *Bartonella* spp., vector—borne pathogens

## Abstract

Deer keds are hematophagous ectoparasites (Diptera: Hippoboscidae) that mainly parasitize Cervidae. These flies are particularly important for animal health due to the occurrence of numerous pathogenic microorganisms. They may also attack humans and their bites may cause allergenic symptoms. The aim of the study was to identify the molecular characteristics of *Borrelia burgdorferi* sensu lato and *Bartonella* spp. pathogens detected in *Lipoptena* spp. sampled both from the hosts and from the environment. For identification of *Bartonella* spp and *B. burgdorferi* s. l., the primers specific to the *rpoB* and *flaB* gene fragments were used, respectively. The overall prevalence of *B. burgdorferi* s.l. DNA in *Lipoptena cervi* was 14.04%, including 14.8% infection in the tested group of winged specimens. The overall prevalence of *Bartonella* spp. was 57.02%. The presence of these bacteria was detected in 53.5% of specimens of *L. cervi* and 75.7% of *L. fortisetosa*. The phylogenetic analysis showed five new haplotypes of the *rpoB* gene of *Bartonella* sp. isolated from *L. cervi/Lipoptena fortisetosa.* We also identified one new haplotype of *B. afzelii* and three haplotypes of *B. burgdorferi* isolated from winged specimens of *L. cervi.* This is the first study to detect the genetic material of *B. burgdorferi* s.l. in *L. cervi* in Poland and the first report on the identification of these bacteria in host-seeking specimens in the environment.

## 1. Introduction

The family Hippoboscidae (Diptera) is a group of obligate hematophagous ectoparasites of mammals and birds, including more than 213 species and 21 genera [1]. In Europe, five *Lipoptena* species have been described, while in Poland, two of them, *Lipoptena cervi* and *Lipoptena fortisetosa*, have been reported so far [2]. Deer keds are common ectoparasites of cervids, but they can attack a wide range of animals, including European bison, horses, cattle, badgers, dogs, and red foxes [2,3]. Deer keds directly affect the condition of the host. Their blood feeding causes anemia, weight loss, itching, and secondary infections resulting from dermatitis lesions [4]. They may also threaten foresters, hunters, and people who visit forest areas, causing skin lesions that evolve after deer ked bites which are painful, often lead to the development of inflammation of the skin, and often also cause allergic reactions [5,6]. Moreover, in deer keds, the presence of the DNA of several pathogens has been described, including bacteria, such as *Bartonella* spp., *Borrelia burgdorferi* sensu lato, *Anaplasma* spp., *Coxiella* spp., *Mycoplasma* spp. *Francisella tularensis*, and *Ehrlichia* spp., protozoa such as *Trypanosoma* spp., and apicomplexan parasites, such as *Babesia* spp. or *Theileria* spp. [7,8,9,10,11].

Bartonellae are small, intracellular Gram-negative bacteria distributed in a wide range of hematophagous arthropods and vertebrates worldwide [12]. About 53 of the *Bartonella* species and three subspecies have been described (https://lpsn.dsmz.de/genus/bartonella; accessed on 12 November 2021). Some of these species have been recognized as potentially zoonotic agents causing human disease with various cardiovascular, neurological, and rheumatological conditions [13]. Blood-feeding arthropods, such as human lice (*Pediculus humanus*), cat fleas (*Ctenocephalides felis*), sand flies (*Lutzomyia verrucarum*), and various hard tick species (*Ixodes* spp., *Dermacentor* spp., as well as *Haemaphysalis* spp.), may be involved in the transmission of *Bartonella* pathogens [7].

In turn, an ethological agent of Lyme borreliosis, the spirochaete *B. burgdorferi* s.l., is a complex of 22 genospecies, of which 11 occur in Europe. Five of them: *Borrelia afzelii*, *Borrelia garinii*, *Borrelia burgdorferii* sensu stricto, *Borrelia spielmanii* and *Borrelia bavariensis*, are associated with human Lyme disease [14,15]. In Europe, the primary vector of *Borrelia* spirochetes is the *Ixodes* spp. tick [16].

This study aimed to determine the presence of *Bartonella* spp. and *B. bugdorferii* s.l. DNA in *Lipoptena* spp. collected from cervids and winged specimens from the environment. Moreover, the study presents the molecular characteristics and phylogenetic analyses of these bacterial pathogens.

## 2. Results

A total of 235 individuals of *Lipoptena* spp. were collected, from which two species were identified: 198 belonging to *L. cervi* (including 27 adults winged) and 37 of *L. fortisetosa* (Table 1).

The overall prevalence of *B. burgdorferi* s.l DNA in *L. cervi* was 14.04% (33/235), including 14.8% (4/27) infection in the tested group of winged specimens. *Borrelia burgdorferi* s.l. DNA was not detected in *L. fortisetosa*. In turn, *Bartonella* spp. was detected in both *Lipoptena* species. The overall prevalence of *Bartonella* spp. was 57.02% (134/235). The presence of these bacteria was detected in 53.5% (106/198) specimens of *L. cervi* and in 75.7% (28/37) of *L. fortisetosa*. Co-infection with two pathogens, *B. burgdorferi* s.l. and *Bartonella* spp., was detected in 23 from a total of 198 (11.61%) *L. cervi.*

**Figure 1:**
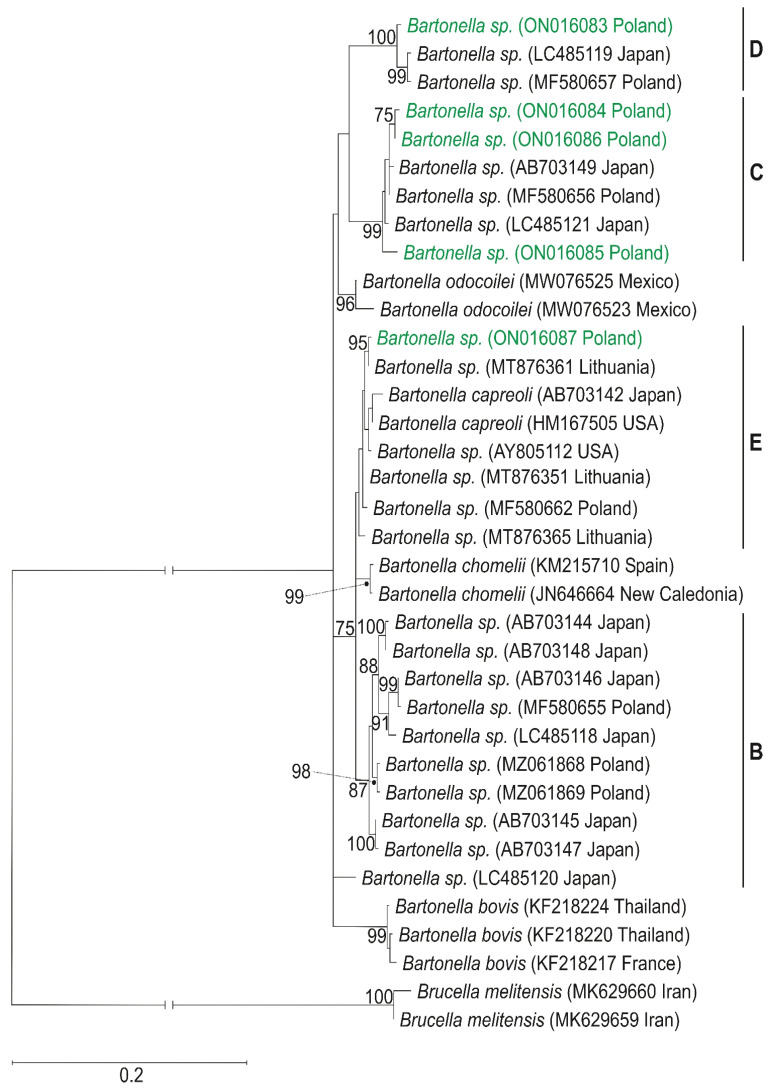
Maximum-likelihood tree computed with the GTR+I+G model of sequence evolution, representing phylogenetic relationships among the sequences of *rpoB* gene for RNA polymerase beta—subunit of *Bartonella* sp. found in Poland (H1–H5, marked in green) and downloaded (H6–H36) from GenBank. Numbers listed at nodes represent percent support for that node from 1000 bootstrap replicates. The tree has been rooted with sequences of *Brucella melitensis*. Lineages B, C, D, E, according to Sato et al. [17,18].

**Figure 2:**
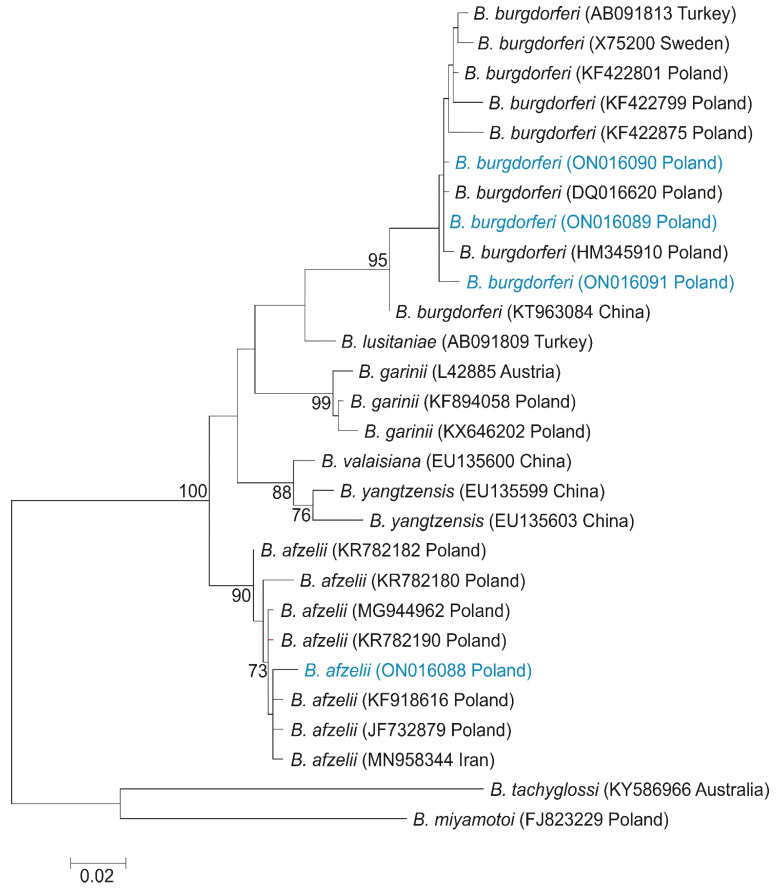
Maximum-likelihood tree computed with the GTR + G model of sequence evolution, representing phylogenetic relationships among the sequences of *flagellin* gene of *Borreliella* sp. found in Poland (H1–H4, marked in blue) and downloaded (H5–H28) from GenBank. Numbers listed at nodes represent percent support for that node from 1000 bootstrap replicates. The tree has been rooted with sequences of *B*. *tachyglossi* and. *B*. *miyamotoi*.

The derived sequences of *Bartonella* (*rpoB* gene) and *Borreliella* (*flaB* gene) species were submitted to the GenBank database (Acc. No. ON016083—ON016087 for *Bartonella* sp., ON016088 for *Borrelia afzelii*, and ON016089—ON016091 for *Borrelia burgdorferi*). The maximum likelihood of phylogenetic reconstructions produced a strong topology (Figure 1 and Figure 2).

**Figure 3:**
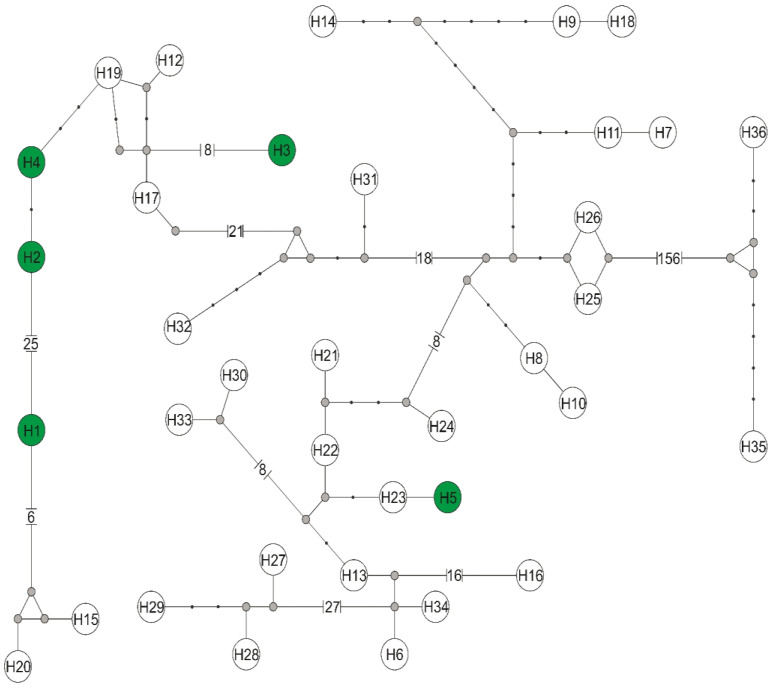
Median-joining network of *rpoB* gene haplotypes for RNA polymerase beta—subunit of the genus *Bartonella*. Haplotypes obtained in this study with the number from H1 to H5 are marked with a green background, while haplotypes downloaded from GenBank have symbols H6–H36 (symbols according to Supplementary files Table S1). Missing haplotypes are indicated by a grey dot.

Among three individuals of *L. cervi* and three individuals of *L*. *fortisetosa,* there were found five haplotypes of the *rpoB* gene of *Bartonella* sp., defined by 53 polymorphic sites, 46 transitions, and 7 transversions. The *rpoB* sequences obtained in the current study were classified into a distinct *Bartonella* phylogenetic lineage named C, D, and E [18], and represented a novel *Bartonella* species. The *rpoB* haplotype H1 belongs to a distinct phylogenetic branch within lineage D, while the phylogenetic lineage C is represented in the current study by haplotypes: H2 (host *L*. *fortisetosa*), H3 (host *L*. *cervi*), and H4 (host *L*. *fortisetosa*). The haplotype H5 belongs to the distinct phylogenetic lineage E. Phylogenetic analyses corroborated the results obtained from the nucleotide network (Figure 3) and confirmed that haplotypes of *Bartonella* derived in this study are divided into three phylogenetic lineages: C (haplotypes H2, H3, and H4), D (haplotype H1), and E (haplotype H5). Haplotype H1 differed by eight mutation steps from haplotypes H15 (GenBank Acc. No. LC485119) found in Japan and H20 (GenBank Acc. No. MF580657) described in Poland. Three and five mutation steps separated haplotypes H2 and H4 in the current study from the Japanese haplotype H19 (GenBank Acc. No. MF580656). Haplotype H3 differed by nine substitutions from H17 (GenBank Acc. No. LC485121) obtained in Japan. Haplotypes H5 and H23 from Lithuania (GenBank Acc. No. MT876361) differed in only one mutation step.

**Figure 4:**
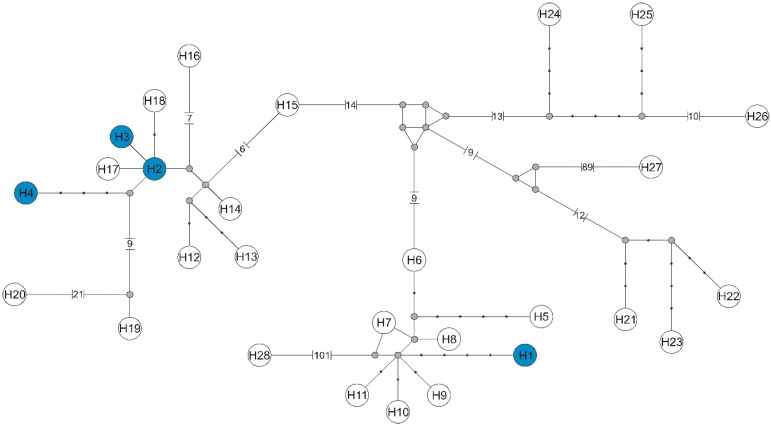
Median-joining network of *flagellin* gene haplotypes of the genus *Borrelia*. Haplotypes found in this study with the number from H1 to H4 are marked with a blue background, while haplotypes downloaded from GenBank have symbols H5–H28 (symbols according to Supplementary files Table S2). Missing haplotypes are indicated by a grey dot.

Among four individuals of *L. cervi*, four haplotypes were found of the *flagellin* gene of *Borrelia* sp. Haplotype H1 belongs to *B*. *afzelii*, while haplotypes H2, H3, and H4 represented *B*. *burgdorferi*, and were defined by six polymorphic sites, four transitions, and two transversions. The haplotype network based on the *flaB* sequences revealed the presence of the same *Borrelia* sp. phylogenetic lineages (Figure 4).

Haplotype H1 belonging to *B*. *afzelii* differed by seven substitutions from haplotypes H7 (GenBank Acc. No. MG944962), H8 (GenBank Acc. No. KR782190), H9 (GenBank Acc. No. JF732879), and H10 (GenBank Acc. No. KF918616) described in Poland, and haplotype H11 (GenBank Accession No. MN958344) found in Iran (Supplementary files Table S2).

Nine and 12 mutation steps separated the H1 haplotypes from H6 (GenBank Acc. No. KR782182) and H5 (GenBank Acc. No. KR782180), also obtained in Poland. Haplotypes H2, H3, and H4 belonging to the *B*. *burgdorferi* differed by at least one substitution from haplotypes H17 (GenBank Acc. No. DQ016620) and H18 (GenBank Acc. No. HM345910), described in Poland

## 3. Discussion

The present study is the first report on the detection of *B. burgdorferi* s.l. DNA in *L. cervi* in Poland. Interestingly, these bacteria were detected in 4 out of 27 winged insects sampled from the environment and specimens collected from cervids. Deer keds have a specific development cycle. These flies are viviparous species, in which the offspring develop into mature third-stage larvae within the uterus of the female. They generate fully-grown larvae that pupate immediately after falling to the ground. When the flies find a suitable host, they shed their wings (then take a blood meal) and remain in the wingless form for the rest of their lives [2,19]. The results obtained in this study may suggest that this bacteria is transferred during the embryonic development of the larvae. Further detailed studies on the parasite-host system are needed to confirm or exclude this hypothesis. The existence of this type of transmission in *Lipoptena* spp., has been shown by De Bruin et al. [20] in the case of *Bartonella schoenbuchensis* and *A. phagocytophilum* in wingless females, developing larvae, and fully developed pupae. The authors indicated vertical transmission of these pathogens from female *L. cervi* to their offspring. In turn, Gałęcki et. al. [11] identified the genetic material of *Bartonella* spp., *Mycoplasma* spp. and *Rickettsia* spp. in winged specimens of *L. fortisetosa* sampled from the environment. The results obtained indicated that *L. fortisetosa* carries the DNA of pathogens, which might be collected through bloodmeal and transferred during the embryonic development of the larvae. Similarly, Korhonen et al. [21] detected *Bartonella* spp. DNA in an unfed adult deer ked. In the future, it would be useful to perform molecular analyzes on several genes, including nuclear and mitochondrial markers, to link the deer ked’s life cycle to the genetic variant.

It is also interesting that *B. burgdorferi* DNA was also detected in deer keds collected from cervids which are incompetent hosts for these pathogens [22]. Buss et al. [8], using PCR, confirmed the presence of *B. burgdorferi* and *A. phagocytophilum* in *L. cervi* removed from white-tailed deer. The prevalence of infection by these pathogens in the tested specimens was 39.50% for *B. burgdorferi* and 29.12% for *A. phagocytophilum*. In results obtained in the current study, *B.* *burgdorferi* was detected in 13.94% of *L. cervi* collected from red deer. In a similar study, Gałęcki et al. [11] identified the genetic material of *Babesia* spp., *Borrelia* spp., and *Theileria* spp. in *L. fortisetosa*, which had had direct contact with cervids. These results may suggest that these insects took blood from the host at an early stage of infection with this spirochete. However, based on the results obtained in this study, we were unable to make such a conclusion.

The phylogenetic analysis based on flagellin gene (*flaB*) sequences of different species of *Borrelia* genus showed the presence of one new haplotype of *B. afzelii* and three haplotypes of *B. burgdorferi*, of which two are newly described. A haplotype of *B*. *afzelii* grouped with haplotypes described in Poland by Wodecka et al. [23] (GenBank Acc. Nos. KF918616 and JF732879), and in Iran by Naddaf et al. [24] (GenBank Acc. No. MN958344), showed a 98.84% similarity with these sequences (Figure 2). On the ML tree, haplotypes of *B. burgdorferi* were grouped with sequences described in Poland by Wodecka et al. [23] (GenBank Acc. No. DQ016620 and HM345910).

In turn, the presence of *Bartonella* spp. in both species, *L. cervi* and *L. fortisetosa,* was confirmed in the current study. The bacteria DNA was found in 57.02% of deer keds collected from cervids. A similarly high percentage (33.3%) of *L. fortisetosa* infected by this bacteria collected from a different animal host in the area of eastern Poland, was shown by Bartosik et al. [25]. Szewczyk et al. [26] showed that the overall prevalence of infection with *Bartonella* spp. was 75.12% among *L. cervi* collected from red deer. Gałęcki et al. [11] detected the DNA of these pathogens in 63.2% of *L. fortisetosa.*

Molecular analysis of an RNA polymerase beta subunit (*rpoB*) gene fragment revealed five new haplotypes of *Bartonella* sp., represented as C, D, and E phylogenetic lineage of this species [17,18]. Haplotype H1 of *Bartonella* sp. (host *L*. *fortisetosa*), representing lineage D, grouped with haplotypes described in Poland by Szewczyk et al. [26] (GenBank Acc. No. MF580657), and in Japan by Sato et al. [18] (GenBank Acc. No. LC485119), revealed a 98.8% similarity (Figure 1). The *rpoB* sequences H2–H4, belonging to the phylogenetics group C, are also grouped with haplotypes described in Japan [17,18] (GenBank Acc. Nos. AB703149 and LC485121, respectively) and in Poland [26] (GenBank Acc. No. MF580656). The haplotype H5 of *Bartonella* sp. found in two different individuals of *L*. *cervi* shared a 99.85% similarity with *Bartonella* sp. from moose blood in Lithuania (GenBank Acc. No. MT876361; unpublished). Haplotype H5 and sequences obtained in Lithuania grouped together with haplotype of *Bartonella* sp., were found in blood from white-tailed deer in the USA [27] (GenBank Acc. No. AY805112). The grouping of the haplotypes identified in our survey with the genetic variants described in the GenBank indicates their close relationship and common origin.

Co-infections of *Lipotena* spp. are also known, but they are rare. Busset et al. [8], in their study, showed co-infection with *B. burgdorferi* s.l. and *A. phagocytophilum* in 6.25% of *L. cervi*. In addition, the genetic material of *Coxiella* spp., *Trypanosoma* spp., *Theileria luwenshuni*, and *T. ovis* have been identified in *L. fortisetosa* [9,10,28]. The results obtained in the present study can confirm the possibility of the occurrence of the presence of more than one pathogen in these insects.

In conclusion, both the results of the present study and the literature data indicate the possibility of the occurrence of various pathogens in *Lipotena* spp. The obtained data indicate that deer keds may potentially harbor both the studied pathogens, and might be an important biological marker for research on their circulation in the environment. However, further detailed studies are necessary to confirm that these bloodsucking insects could be treated as their potential biological vector and/or reservoir, and their potential transmission by deer keds should be assessed in accordance with Koch’s postulates [29,30].

## 4. Materials and Method

### 4.1. Field Work

The study was carried out in the Piska Forest (53°46′ N, 21°27′ E) and the Białowieża Primeval Forest (52°42′ N, 23°52′ E) located in north-eastern Poland. Deer keds were collected manually using tweezers from the fur of red deer during the autumn hunting season. In total, 208 *Lipoptena* spp. were collected from five hunted red deer in the Białowieża Primeval Forest and from 12 hunted red deer in the Piska Forest. The animals were culled in accordance with the Annual Hunting Plans in selected hunting circles operating in the studied macroregion, during hunting periods indicated in the Regulation of the Minister of the Environment of 16 March 2005 on the determination of hunting periods for game animals (*Journal of Laws 2005*, No. 48, item 459). Insects (27 specimens) were also collected from vegetation in autumn using an entomological net and after landing on clothing in the Białowieża Primeval Forest. The collected material was preserved in plastic sample tubes containing 70% ethyl alcohol. Species identification of the collected specimens was carried out using taxonomic keys according to Borowiec [2], Andreani et al. [31], and Salvetti et al. [32] under a stereoscopic microscope (OPTA—TECH, Warsaw, Poland).

### 4.2. PCR Detection of Bartonella spp. and B. burgdorferi s.l.

The DNA from each fly was extracted using the AX Tissue Mini kit (A&A Biotechnology, Gdynia, Poland) according to the manufacturer’s protocol. The DNA was measured spectrophotometrically in a nanospectrophotometer PEARL (Implen, Germany) and then frozen to −20 °C for further molecular study. To detect *Bartonella* sp., a 200 ng DNA template and a pair of primers—1400F/2300R—were used to amplify an 850 base pairs (bp) fragment of the *rpo*B gene [33]. PCR reactions were conducted according to Paziewska et al. [34]. For the reaction mixture, RUN *Taq* polymerase (A&A Biotechnology, Gdynia, Poland) was used. In turn, *B. burgdorferi* s.l. was detected in insects with the use of two pairs of primers specific to the *flaB* gene fragment, as previously described [23,35]. For amplification, a 200 ng DNA template was used. In turn, for re—amplification, 1 µL of the amplification product was used. DFS—Plus DNA Taq Polymerase (GeneOn, Ludwigshafen am Rhein, Germany) was used for both reactions. The presence of 824 bp (*rpoB* gene) and 605 bp (*flaB* gene) reaction products was considered positive. PCR and nested PCR products were visualized on 1% and 2% ethidium—bromide—stained agarose gels. Gels were visualized using ChemiDoc, MP Lab software (Imagine, BioRad, Hercules, Clearwater, FL, USA) or Omega 10 (UltraLum, Berlin, CT, USA) and TotalLab software (TotalLab, Newcastle upon Tyne, UK). The positive products of PCR and nested PCR were purified using a QIAEX II Gel extraction kit (Qiagen, Hilden, Germany) or Agarose—Out DNA Purification Kit (EURx, Gdańsk, Poland), and sequenced by Genomed (Warsaw, Poland).

### 4.3. Phylogenetic Analysis

The resulting six sequences of the *rpoB* gene for the RNA polymerase beta subunit of *Bartonella* sp. and four sequences of *flagellin* gene (*flaB*) of *Borrelia* genus were aligned and revised manually in BioEdit v 7.0.4 [36]. To determine bacteria species, the DNA sequences were compared with the GenBank references (Supplementary files) by BLAST (http://www.ncbi.nlm.nih.gov/; accessed on 14 November 2021).

To test the phylogenetic relationships among the obtained haplotypes of *rpoB* and *flaB* genes and sequences downloaded from GenBank (Supplementary files), phylogenetic trees were constructed using a maximum-likelihood (ML) algorithm in MEGA6 v.06 [37] with 1000 bootstrap replicates to assess the tree node support. Also used were additional sequences of *Brucella melitensis* (GenBank Acc. No. MK629659 and MK629660 for *rpoB* gene) and *Borrelia* species (*B*. *tachyglossi* (GenBank Acc. No. KY586966) and *B*. *miyamotoi* (GenBank Acc. No. FJ823229), both for *flaB* gene) downloaded as trees outgroups. In the phylogenetic analyses, a nucleotide substitution model was used, determined under the Akaike information criterion [38] implemented in jModelTest v. 0.1.1 [39]. The GTR+I+G model was selected as the best—fitting model for *rpoB* gene sequences, while for *flagellin* gene sequences, the GTR+G model was chosen. Also calculated and visualized were the relationships among haplotypes and sequences of *rpoB* and *flaB* gene downloaded from GenBank, by constructing a haplotype network using the median—joining method available in Network v. 10.2.0.0 (http://www.fluxus—engineering.com; accessed on 10 December 2021).

## Figures and Tables

**Table 1:** Specimens of *Lipoptena cervi* and *Lipoptena fortisetosa* analysed for *Bartonella* spp. and *Borrelia burgdorferi* s.l. infection.

Processed Deer Ked	Location	*Bartonella* spp.	*Borrelia burgdorferi* s.l.
*Lipoptena cervi*	Białowieża Primeval Forest	13/25 ♀	12/25 ♀
		10/28 ♂	8/28 ♂
	Piska Forest	44/75 ♀	10/75 ♀
		39/70 ♂	3/70 ♂
*Lipoptena fortisetosa*	Białowieża Primeval Forest	2/8 ♀	0/8 ♀
		1/2 ♂	0/2 ♂
	Piska Forest	15/16 ♀	0/16 ♀
		10/11 ♂	0/11 ♂

## Data Availability

Raw sequencing data are available on the National Center for Biotechnology Information under projectaccession number ON016083—ON016087 (for *Bartonella* sp.) ON016088 (for *Borrelia afzelii*), and ON016089—ON016091 (for *Borrelia burgdorferi*) (https://www.ncbi.nlm.nih.gov/nuccore/, accessed on 22 September 2022).

## Supplementary Materials

The following are available online at https://www.mdpi.com/article/10.3390/pathogens11101111/s1, Table S1: List of species and GenBank accession numbers of their RNA polymerase beta subunit (*rpoB*) gene sequences used in the network phylogenetic analysis (Figure 1 and Figure 3). Table S2: List of species and GenBank accession numbers of their flagellin gene (*fla B*) sequences used in the network phylogenetic analysis (Figure 2 and Figure 4). References [40,41,42,43,44,45,46,47,48,49,50] are cited in the supplementary materials.
