# Supplementary material for: The New Haplotypes of Bartonella spp. and Borrelia burgdorferi Sensu Lato Identified in Lipoptena spp. (Diptera: Hippoboscidae) Collected in the Areas of North-Eastern Poland
Source: Pathogens. 2022 Sep 28;11(10):1111. doi: 10.3390/pathogens11101111 (PMC9611934; doi:10.3390/pathogens11101111)
Supplement: Supplementary file 1 [file pathogens-11-01111-s001.zip › Table S1-suplementaryfile.pdf]

Table S1. List of species and GenBank accession numbers of their RNA polymerase beta subunit (r*poB*) gene sequences used in the network phylogenetic analysis (Figure 1 and 3).

**Symbol of haplotype**	**Scientific name**	**GenBank accession number**	**Sequence references**
H1	*Bartonella* sp.	ON016083	This study
H2	*Bartonella* sp.	ON016084	This study
H3	*Bartonella* sp.	ON016085	This study
H4	*Bartonella* sp.	ON016086	This study
H5	*Bartonella* sp.	ON016087	This study
H6	*Bartonella capreoli*	AB703142	[17]
H7	*Bartonella* sp.	AB703144	[17]
H8	*Bartonella* sp.	AB703145	[17]
H9	*Bartonella* sp.	AB703146	[17]
H10	*Bartonella* sp.	AB703147	[17]
H11	*Bartonella* sp.	AB703148	[17]
H12	*Bartonella* sp.	AB703149	[17]
H13	*Bartonella* sp.	AY805112	[27]
H14	*Bartonella* sp.	LC485118	[18]
H15	*Bartonella* sp.	LC485119	[18]
H16	*Bartonella* sp.	LC485120	[18]
H17	*Bartonella* sp.	LC485121	[18]
H18	*Bartonella* sp.	MF580655	[26]
H19	*Bartonella* sp.	MF580656	[26]
H20	*Bartonella* sp.	MF580657	[26]
H21	*Bartonella* sp.	MF580662	[26]
H22	*Bartonella* sp.	MT876351	Unpublished
H23	*Bartonella* sp.	MT876361	Unpublished
H24	*Bartonella* sp.	MT876365	Unpublished
H25	*Bartonella* sp.	MZ061868	[25]
H26	*Bartonella* sp.	MZ061869	[25]
H27	*Bartonella bovis*	KF218224	[40]
H28	*Bartonella bovis*	KF218220	[40]
H29	*Bartonella bovis*	KF218217	[40]
H30	*Bartonella chomelii*	KM215710	[41]
H31	*Candidatus Bartonella odocoilei*	MW076525	[42]
H32	*Candidatus Bartonella odocoilei*	MW076523	[42]
H33	*Bartonella chomelii*	JN646664	[43]
H34	*Bartonella capreoli*	HM167505	[40]
H35	*Brucella melitensis*	MK629660	[44]
H36	*Brucella melitensis*	MK629659	[44]
