# Supplementary material for: The New Haplotypes of Bartonella spp. and Borrelia burgdorferi Sensu Lato Identified in Lipoptena spp. (Diptera: Hippoboscidae) Collected in the Areas of North-Eastern Poland
Source: Pathogens. 2022 Sep 28;11(10):1111. doi: 10.3390/pathogens11101111 (PMC9611934; doi:10.3390/pathogens11101111)
Supplement: Supplementary file 1 [file pathogens-11-01111-s001.zip › Table S2-suplementaryfile.pdf]

Table S2. List of species and GenBank accession numbers of their flagellin gene (*fla B*) sequences used in the network phylogenetic analysis (Figure 2 and 4).

Symbol of haplotype	Scientific name	GenBank accession number	Sequence references
H1	*Borrelia afzelii*	ON016088	This study
H2	*Borrelia burgdorferi*	ON016089<br>HM345911	This study<br>[45]
H3	*Borrelia burgdorferi*	ON016090	This study
H4	*Borrelia burgdorferi*	ON016091	This study
H5	*Borrelia afzelii*	KR782180	Unpublished
H6	*Borrelia afzelii*	KR782182	Unpublished
H7	*Borrelia afzelii*	MG944962	Unpublished
H8	*Borrelia afzelii*	KR782190	Unpublished
H9	*Borrelia afzelii*	JF732879	Unpublished
H10	*Borrelia afzelii*	KF918616	Unpublished
H11	*Borrelia afzelii*	MN958344	[46]
H12	*Borrelia burgdorferi*	AB091813	[47]
H13	*Borrelia burgdorferi*	X75200	[48]
H14	*Borrelia burgdorferi*	KF422801	[49]
H15	*Borrelia burgdorferi*	KF422799	Unpublished
H16	*Borrelia burgdorferi*	KF422875	[49]
H17	*Borreliea burgdorferi*	DQ016620	[45]
H18	*Borrelia burgdorferi*	HM345910	[45]
H19	*Borrelia burgdorferi*	KT963084	Unpublished
H20	*Borrelia lusitaniae*	AB091809	[47]
H21	*Borrelia garinii*	L42885	[50]
H22	*Borrelia garinii*	KF894058	Unpublished
H23	*Borrelia garinii*	KX646202	Unpublished
H24	*Borrelia valaisiana*	EU135600	[51]
H25	*Borrelia yangtzensis*	EU135599	[51]
H26	*Borrelia yangtzensis*	EU135603	[51]
H27	*Candidatus Borrelia tachyglossi*	KY586966	[52]
H28	*Borrelia miyamotoi*	FJ823229	[45]

References:

40. Bai, Y.; Malania, L.; Alvarez Castillo, D.; Moran, D.; Boonmar, S.; Chanlun, A.; Suksawa, F.; Maruyama, S.; Knobel, D.; Kosoy, M. Global distribution of Bartonella infections in domestic bovine and characterization of Bartonella bovis strains using multi-locus sequence typing. PLoS One, **2013**, 21:e80894. doi: 10.1371/journal.pone.0080894.
41. Antequera-Gómez, M.L.; Lozano-Almendral, L.; Barandika, J.F.; González-Martín-Niño, R.M.; Rodríguez-Moreno, I.; García-Pérez, A.L.; Gil, H. Bartonella chomelii is the most frequent species infecting cattle grazing in communal mountain pastures in Spain. Appl Environ Microbiol, **2015** 81:623-9. doi: 10.1128/AEM.03159-14.
42. Lozano-Sardaneta, Y.N.; Blum-Domínguez, S.; Huerta, H.; Tamay-Segovia, P.; Fernández-Figueroa, E.A.; Becker, I.; Sánchez-Montes, S. Detection of Candidatus Bartonella odocoilei n. sp. in Lipoptena mazamae associated with white-tailed deer in Campeche, Mexico. Med Vet Entomol. **2021,** 35:652-657. doi: 10.1111/mve.12536.
43. Mediannikov, O.; Davoust, B.; Cabre, O.; Rolain, J.M.; Raoult, D. Bartonellae in animals and vectors in New Caledonia. Comp Immunol Microbiol Infect Dis. **2011**, 34:497-501. doi: 10.1016/j.cimid.2011.09.002.
44. Bazrgari, N.; Garoosi, G.A.; Dadar, M. Genetic Diversity and Phylogenetic Relationship of Clinical Isolates of Brucella melitensis Based on Gene Polymorphism of β Subunit of RNA Polymerase (rpoB) Gene in Iran**.** Iran J Med Microbiol, **2020**, 14: 425-440.
45. Wodecka, B. flaB gene as a molecular marker for distinct identification of Borrelia species in environmental samples by the PCR-restriction fragment length polymorphism method. Appl Environ Microbiol. **2011**, 77:7088-92. doi: 10.1128/AEM.05437-11.
46. Naddaf, S.R.; Mahmoudi, A.; Ghasemi, A.; Rohani, M.; Mohammadi, A.; Ziapour, S.P.; Nemati, A.H.; Mostafav,i E. Infection of hard ticks in the Caspian Sea littoral of Iran with Lyme borreliosis and relapsing fever borreliae. Ticks Tick Borne Dis. **2020**, 11:101500. doi: 10.1016/j.ttbdis.2020.101500.
47. Güner, E.S.; Hashimoto, N.; Takada, N.; Kaneda, K.; Imai, Y.; Masuzawa, T. First isolation and characterization of Borrelia burgdorferi sensu lato strains from Ixodes ricinus ticks in Turkey. J Med Microbiol. **2003**, 52:807-813. doi: 10.1099/jmm.0.05205-0.
48. Noppa, L.; Burman, N.; Sadziene, A.; Barbour, A.G.; Bergström, S. Expression of the flagellin gene in Borrelia is controlled by an alternative sigma factor. Microbiology (Reading). **1995**, 141:85-93. doi: 10.1099/00221287-141-1-85.
49. Wodecka. B.; Skotarczak, B. Identification of host blood-meal sources and Borrelia in field-collected Ixodes ricinus ticks in north-western Poland. Ann Agric Environ Med. **2016**, 23:59-63. doi:10.5604/12321966.1196853.
50. Livey, I.; Gibbs, C.P.; Schuster, R.; Dorner, F. Evidence for lateral transfer and recombination in OspC variation in Lyme disease Borrelia. Mol Microbiol. **1995** 18:257-69. doi: 10.1111/j.1365-2958.1995.mmi_18020257.x.
51. Chu, C.Y.; Liu, W.; Jiang, B.G.; Wang, D.M.; Jiang, W.J.; Zhao, Q.M.; Zhang, P.H.; Wang, Z.X.; Tang, G.P.;, Yang, H.; Cao, W.C. Novel genospecies of Borrelia burgdorferi sensu lato from rodents and ticks in southwestern China. J Clin Microbiol. **2008,** 46:3130-3. doi: 10.1128/JCM.01195-08.
52. Loh, S.M.; Gillett, A.;Ryan, U.; Irwin, P.; Oskam, C. Molecular characterization of 'Candidatus Borrelia tachyglossi' (family Spirochaetaceae) in echidna ticks, Bothriocroton concolor. Int J Syst Evol Microbiol. **2017,** 67:1075-1080. doi: 10.1099/ijsem.0.001929.
